# Primary malignant melanoma of the esophagus with multiple lymph node metastases: A case report and literature review

**DOI:** 10.1097/MD.0000000000018573

**Published:** 2020-05-29

**Authors:** Kenichi Iwasaki, Yoshihiro Ota, Erika Yamada, Kosuke Takahashi, Takafumi Watanabe, Yosuke Makuuchi, Takeshi Suda, Yoshiaki Osaka, Akiyoshi Seshimo, Kenji Katsumata, Akihiko Tsuchida

**Affiliations:** Department of Gastrointestinal and Pediatric Surgery, Tokyo Medical University, Tokyo, Japan.

**Keywords:** esophageal malignant melanoma, esophagus, lymph node metastasis, prognosis, survival

## Abstract

**Rationale:**

Primary malignant melanoma of the esophagus (PMME) is a very rare malignancy accounting for only 0.1% to 0.2% of all malignant esophageal lesions. Presently, there are no standard strategies or clear guidelines for PMME treatment.

**Patient concerns:**

Herein, we report a patient who had PMME with multiple lymph node metastases (LNMs) who was treated successfully by esophagectomy. In March 2018, a 74-year-old man with symptoms of continuous dysphagia was referred to our hospital.

**Diagnosis:**

Upper gastrointestinal endoscopic examination revealed melanin pigmentation in the middle thoracic esophagus and a pigmented polypoid mass in the lower esophagus. Histopathological examination of the endoscopic biopsy specimen revealed malignant melanoma. Contrast-enhanced computed tomography showed a 3 cm tumor lesion with several enlarged lymph nodes without distant metastasis. The preoperative diagnosis based on the TNM classification was cT2N2M0 stage III.

**Interventions:**

The patient underwent esophagectomy with lymph node dissection.

**Outcomes:**

Histopathological examination showed that the tumor extended to the submucosal layer of the esophageal wall, with multiple LNMs. Although multiple LNMs were detected, computed tomography scan 15 months after surgery showed no recurrence. Additionally, we analyzed the relationship between the overall survival and the clinicopathological factors including LNMs in 48 previously reported cases of PMME that were surgically treated.

**Lessons:**

To our knowledge, this is the first report on the effect of LNMs on the prognosis of PMME patients. The analysis revealed the prognostic value of the TNM stage. Early tumor detection and esophagectomy with lymph node dissection may play as key factors for achieving a better overall survival of PMME patients.

## 1 Introduction

Primary malignant melanoma of the esophagus (PMME) is a very rare malignancy accounting for only 0.1% to 0.2% of all malignant esophageal lesions.^[1–3]^ PMME is highly aggressive with a high potential for metastasis. Almost half of PMME patients have distant metastasis upon diagnosis, and the 5-year survival rate is between 2.2% and 37.5%.^[4–7]^ Standard treatment strategies with strong evidence of success have not yet been established, and radical tumor resection remains the mainstream treatment. Although lymph node involvement is the most important prognostic factor in esophageal cancer,^[8]^ only few reports have suggested that lymph node metastases (LNMs) could be a survival predictor for PMME patients.^[9,10]^ Herein, we report a patient who had PMME with multiple LNMs who was successfully treated by radical resection without recurrence.

## 2 Case report

**Figure 1:**
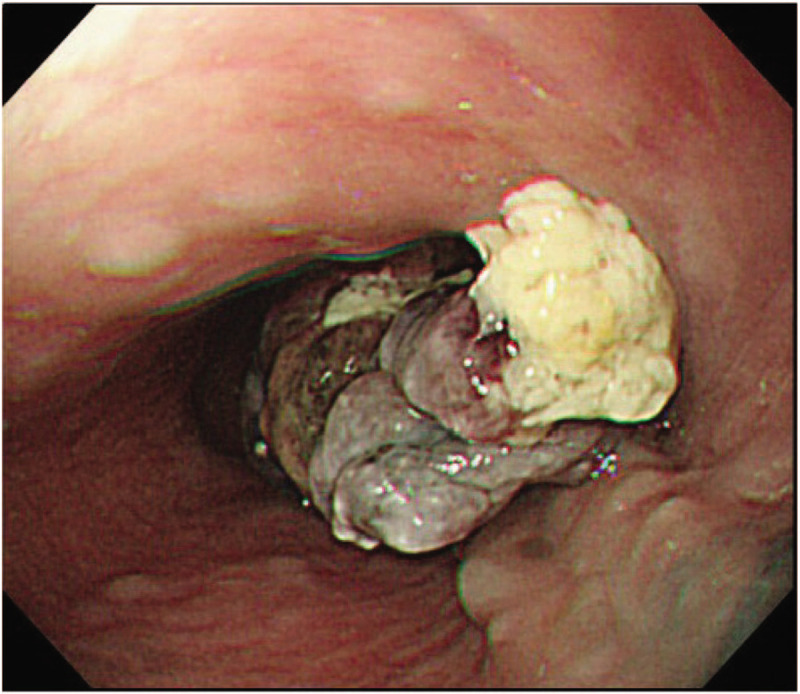
Upper gastrointestinal endoscopy revealed a pigmented mucosa (arrow) located 33 cm from the incisor tooth, and a pigmented polypoid mass (arrowhead) 37 to 42 cm from the incisor tooth.

A 74-year-old man was admitted to our hospital in March 2018 with symptoms of continuous dysphagia. Upper gastrointestinal endoscopy revealed a pigmented mucosa 33 cm from the incisor tooth, and a pigmented polypoid mass 37 to 42 cm from the incisor tooth (Fig. 1).

**Figure 2:**
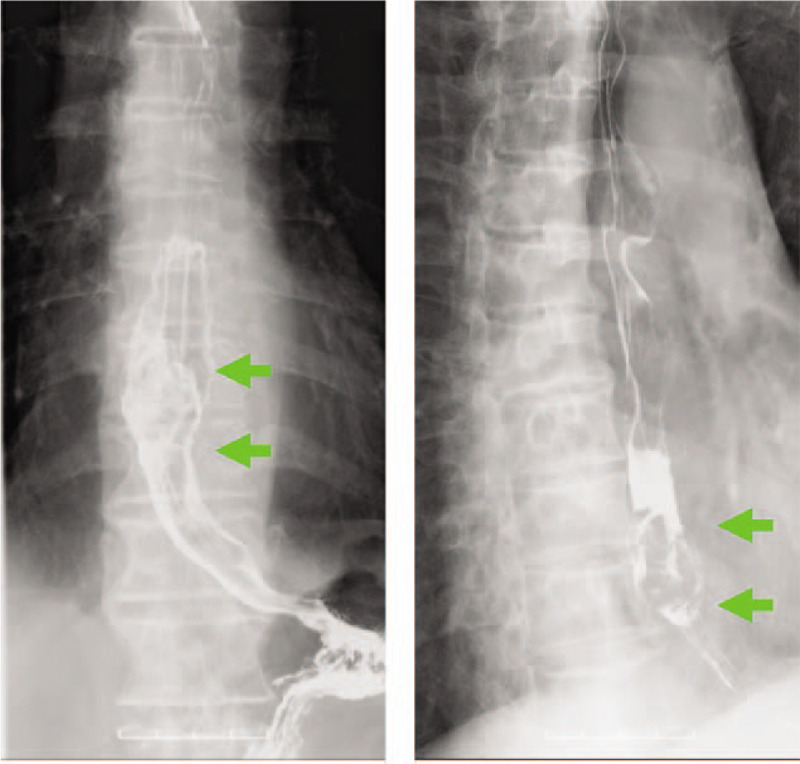
Esophagography showed a 30 mm tumor on the lower thoracic esophageal wall (green arrow).

Histopathological examination of the biopsied tumor showed proliferation of malignant spindle cells with melanin pigmentation, confirming a diagnosis of malignant melanoma. Esophagography showed the main tumor as a 30 mm mass on the lower thoracic esophageal wall (Fig. 2).

**Figure 3:**
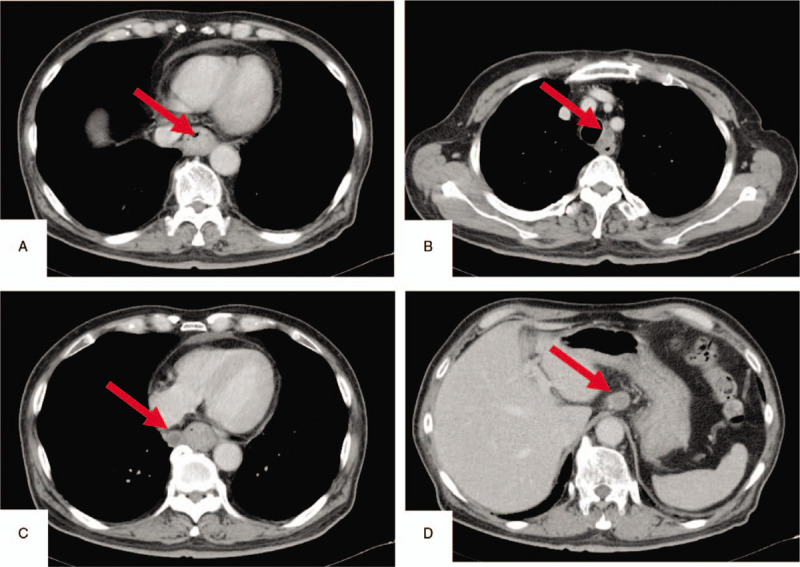
a-d Contrast-enhanced computed tomography showed the tumor occupying the lumen of the lower thoracic esophagus (**a**). Computed tomography also revealed enlarged lymph nodes, namely, the left upper paratracheal (**b**), lower paraesophageal (**c**), and lesser curvature (**d**) lymph nodes.

Contrast-enhanced computed tomography (CT) showed the tumor occupying the lumen of the lower thoracic esophagus without any wall thickening. The CT also revealed enlarged lymph nodes, namely, the left upper paratracheal, lower paraesophageal, and lesser curvature lymph nodes (Fig. 3 a–d). The preoperative diagnosis based on the TNM classification (American Joint Committee on Cancer staging manual ^[11]^) was cT2N2M0 stage III.

The patient underwent minimally invasive video-assisted esophagectomy with 3-field lymph node dissection. The gastric conduit was prepared by hand-assisted laparoscopic surgery and raised by the retrosternal route. The postoperative course was uncomplicated with the patient showing a smooth recovery.

**Figure 4:**
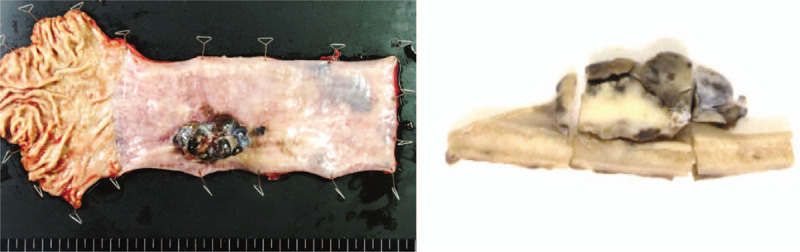
The resected specimen showed a 70 × 50 × 23 mm tumor (arrow). The cut surface of the tumor was irregularly mixed with black and gray material (arrowhead).

The resected tumor was located in the lower thoracic esophagus. The tumor measured 70 × 50 × 23 mm, and the cut surface of the tumor showed an irregularly mixed black and gray material (Fig. 4).

**Figure 5:**
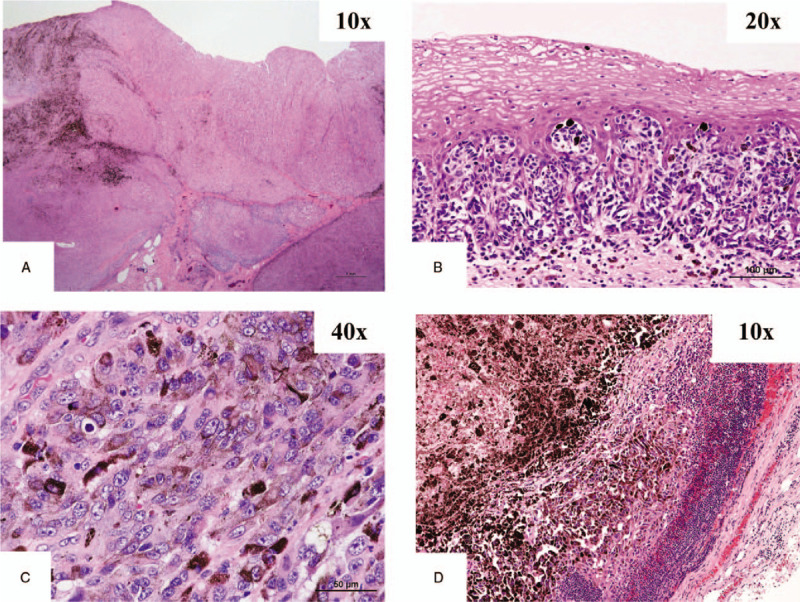
a-d **a**. Histopathological findings of the resected specimen revealed that the surface of the tumor was proliferating as a protruding lesion, partially showing melanin pigmentation (arrow). The tumor cells invaded deeply into the superficial layer of the submucosal layer (arrowhead). **b.** Melanoma cells extended into the mucosal layer forming small phlegmons (arrow). **c.** High magnification showed enlarged spindle-shaped cells (arrow) containing melanin granule (arrowhead). **d.** Proliferating cells (arrow) corresponding to malignant melanoma were evident in the metastatic lymph nodes.

Histopathological findings of the resected specimen revealed that the surface of the tumor was proliferating as a protruding lesion, partially showing melanin pigmentation. The tumor cells invaded deeply into the superficial layer of the submucosal layer (Fig. 5a). Melanoma cells extended into the mucosal layer forming small phlegmons (Fig. 5b). High magnification showed enlarged spindle-shaped cells containing melanin granule (Fig. 5c). Proliferating cells corresponding to malignant melanoma were evident in the metastatic lymph nodes (Fig. 5d).

The pathological TNM classification was malignant melanoma of the esophagus, T1bN3M0 stage IVa. Although multiple LNMs were detected, CT scan 15 months after surgery showed no recurrence.

## 3 Discussion

Malignant melanoma originating from the esophagus (i.e., PMME) is a rare malignancy accounting for only 0.1% to 0.2% of malignant esophageal lesions.^[1–3]^ The disease is more common among the elderly aged 50 to 60 years, occurs mostly in the middle and lower segments of the esophagus, and with an approximate male-to-female ratio of 3:1.^[12]^ The prognosis of PMME is extremely poor. Sabanathan et al reported a postsurgical 1-year survival rate of 35% and a 5-year survival rate of 4% in 1989.^[13]^ In their series of 25 patients who underwent surgery from 1989 to 2000, Volpin et al reported a 5-year survival rate of 37%.^[4]^ More recently, postsurgical 1- and 5-year survival rates of 56.9% and 26.3%, respectively, were reported by Makuuchi et al in 2015.^[14]^ Despite the poor prognosis of PMME, there is still no consensus regarding its standard management. Surgical resection with lymph node dissection remains the most common treatment for PMME. Although some authors have reported the efficacy of neoadjuvant and adjuvant chemotherapies,^[15,16]^ the benefits from these therapies remain controversial and unclear.

Notably, the number of documented PMME cases is extremely limited.^[3,6]^ Our PubMed search identified only 133 surgically resected PMME cases described in 74 reports from 1999 to 2019. We made a questionnaire survey to obtain more details of these 74 reports which included age, gender, tumor size, location, TNM stage, and chemotherapy. The return rate of the questionnaire was 43.2% (48 cases). The clinicopathological characteristics of the 48 PMME patients are summarized in Table 1. The results of the Cox regression analysis of the prognosis factors related to PMME patient survival are shown in Table 2. Cases with insufficient data were excluded from the analysis.

**Table 1:**
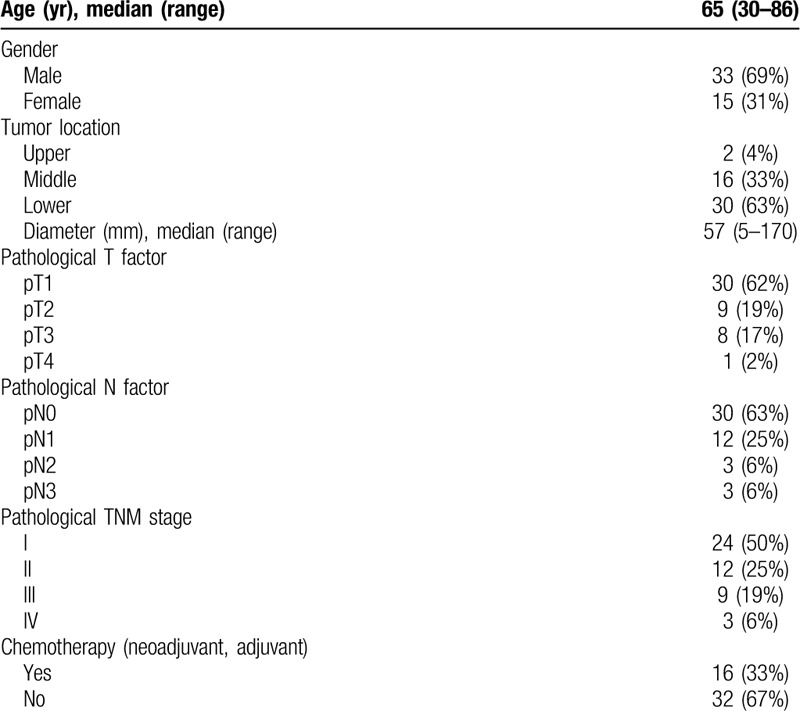
Summary of patient and tumor characteristics (n = 48).

**Table 2:**
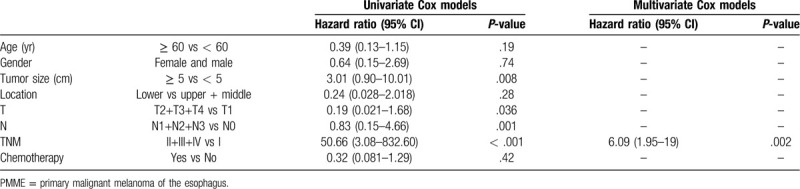
Cox regression analysis of prognosis factors related to PMME patient survival.

The average diameter of the tumor was 5.7 ± 3.8 cm. The majority of the tumors were located in the middle [16 (33%)] or lower [30 (63%)] segment of the esophagus. This finding was consistent with previous reports.^[5,16,6]^ Histopathological examination revealed that the numbers of patients with tumor extension to the mucosa (T1a), submucosal layer (T1b), deep muscular layer, fibrous membrane, and beyond the serosa were 5 (10%), 25 (52%), 9 (19%), 8 (17), and 1 (2%), respectively. The median survival time after surgery was 17.5 months, and the 1-year and 5-year postesophagectomy survival rates were 76.9% and 51.7%, respectively.

To analyze the relationship between the overall survival and the clinicopathological factors, the log-rank test was performed for each factor (Table 2). Univariate Cox regression analysis showed that the tumor size (≥ 5 cm), tumor invasion (deeper than T2), LNMs, and TNM stage were significantly associated with the prognosis of PMME patients. In particular, stage II, III patients were associated with a significantly shorter postoperative OS than stage I patients (*P* < .001). Multivariable Cox regression analysis revealed that the TNM stage was the only independent prognostic factor for PMME patients [HR (95% CI): 6.085 (1.949–19), *P* = .002]. There was no significant difference in the OS between the patients with LNMs and the patients without LNMs (*P* = .82). Considering the highly aggressive biological behavior of this disease, these results strongly suggest that early detection is critical to achieve a better overall survival rate for PMME patients.

The validity of the present analysis has also some limitations. Selection bias may not have been completely excluded as nearly 60% of the PMME patients who underwent surgery were not analyzed owing to the low response rate of the questionnaire survey. The slightly higher 1-year and 5-year OS rates than the previous reports may be due to the high percentage (50%) of stage I patients in the group. Therefore, more cases with complete and accurate information are needed to arrive at a more definitive conclusion.

## 4 Conclusion

We report a patient who had PMME with multiple LNMs who was successfully treated by esophagectomy. Analysis of 48 reported cases of PMME treated by surgery revealed the prognostic value of the TNM stage. Early tumor detection and esophagectomy with lymph node dissection may play as key factors for achieving a better OS of PMME patients.

### 4.2 Statistical analysis

Quantitative data were expressed as mean ± standard deviation (SD). The log-rank test was used to analyze the correlation between the clinicopathological factors and the survival of PMME patients. Univariate and Multivariate Cox regression analyses using a stepwise approach were performed to identify independent prognostic factors for PMME patients. The selected covariables included age, gender, tumor location, chemotherapy, T stage, LNMs, and TNM stage. Statistical analysis was performed using SPSS 13.0 software. A *P*-value of < .05 was considered to indicate a statistically significant difference.

## Acknowledgment

We thank Dr Edward Barroga (https://orcid.org/0000-0002-8920-2607), Medical Editor and Professor of Academic Writing at St. Luke's International University, for editing the manuscript.

## Author contributions

**Conceptualization:** Kenichi Iwasaki, Yoshihiro Ota, Yoshiaki Osaka, Akiyoshi Seshimo, Kenji Katsumata, Akihiko Tsuchida.

**Data curation:** Erika Yamada, Kosuke Takahashi, Takafumi Watanabe, Yosuke Makuuchi, Takeshi Suda.

**Formal analysis:** Akihiko Tsuchida.

**Writing – original draft:** Kenichi Iwasaki.

**Writing – review & editing:** Kenichi Iwasaki.

## References

[1] McCormack PM Nascimento AG Bains MS. Primary melanocarcinoma of the esophagus. Clin Bull 1979;9:162–4. 540421

[2] Caldwell CB Bains MS Burt M. Unusual malignant neoplasms of the esophagus. Oat cell carcinoma, melanoma, and sarcoma. J Thorac Cardiovasc Surg 1991;101:100–7. 1702494

[3] Bisceglia M Perri F Tucci A. Primary malignant melanoma of the esophagus: a clinicopathologic study of a case with comprehensive literature review. Adv Anat Pathol 2011;18:235–52. 21490441 10.1097/PAP.0b013e318216b99b

[4] Volpin E Sauvanet A Couvelard A. Primary malignant melanoma of the esophagus: a case report and review of the literature. Dis Esophagus 2002;15:244–9. 12444999 10.1046/j.1442-2050.2002.00237.x

[5] Iwanuma Y Tomita N Amano T. Current status of primary malignant melanoma of the esophagus: clinical features, pathology, management and prognosis. J Gastroenterol 2012;47:21–8. 22048255 10.1007/s00535-011-0490-y

[6] Zheng J Mo H Ma S. Clinicopathological findings of primary esophageal malignant melanoma: report of six cases and review of literature. Int J Clin Exp Pathol 2014;7:7230–5. 25400820 PMC4230078

[7] Sabat J Mannan R Legasto A. Long-term survivor of primary malignant melanoma of the esophagus treated with surgical resection. Int J Surg Case Rep 2015;6C:182–5. 25543881 10.1016/j.ijscr.2014.12.016 PMC4334882

[8] Lerut T Coosemans W Decker G. Cancer of the esophagus and gastro-esophageal junction: potentially curative therapies. Surg Oncol 2001;10:113–22. 11750230 10.1016/s0960-7404(01)00027-5

[9] Gao S Li J Feng X. Characteristics and surgical outcomes for primary malignant melanoma of the esophagus. Sci Rep 2016;6:23804. 27033424 10.1038/srep23804 PMC4817120

[10] Yamaguchi T Shioaki Y Koide K. A case of primary malignant melanoma of the esophagus and analysis of 193 patients in Japan. Nihon Shokakibyo Gakkai Zasshi 2004;10:1087–94. 15529781

[11] Thomas WR Deepa TP Eugene H. Blackstone. 8th edition AJCC/UICC staging of cancers of the esophagus and esophagogastric junction: application to clinical practice. Ann Cardiothorac Surg 2017;6:119–30. 28447000 10.21037/acs.2017.03.14 PMC5387145

[12] Chalkiadakis G Wihlm JM Morand G. Primary malignant melanoma of the esophagus. Ann Thorac Surg 1985;80:417–20. 10.1016/s0003-4975(10)61963-7 3994450

[13] Sabanathan S Eng J Pradhan GN. Primary malignant melanoma of the esophagus. Am J Gastroenterol 1989;84:1475–81. 2688398

[14] Hiroyasu M Kaiyo T Akio Y. Esophageal malignant melanoma: analysis of 134 cases collected by the Japan Esophageal Society. Esophagus 2015;12:158–69.

[15] Yu H Huang XY Li Y. Primary malignant melanoma of the esophagus: a study of clinical features, pathology, management and prognosis. Dis Esophagus 2011;24:109–13. 21040150 10.1111/j.1442-2050.2010.01111.x

[16] Yano M Shiozaki H Murata A. Primary malignant melanoma of the esophagus associated with adenocarcinoma of the lung. Surg Today 1998;28:405–8. 9590706 10.1007/s005950050150

